# The Present Status of Gall-Bladder Surgery

**Published:** 1914-08

**Authors:** R. R. Kime

**Affiliations:** Atlanta, Ga.


### THE PRESENT STATUS OF GALL-BLADDER SURGERY.

BY R. R. KIME, M. D., F. A. C. S., ATLANTA, GA.

Surgery of the gall-bladder, appendix, and stomach opens us a vast field for study, investigation and careful diagnoses.

It also opens up a wide territory for surgical exploitation and snap-shot diagnosis with consequent needless surgery.

Formerly every pain in the "belly" meant indigestion or gastralgia, now it means surgical operation.

Later on people will be taught HOW TO LIVE and WHAT TO EAT, then there will be less surgery and general medicine will be in the ascendency.

As an illustration—Sanitation and Hygiene will prevent many cases of typhoid fever, the Sensitized Bacterins will prevent and cure many more, while the physician of the future will feel it his duty to prevent gall-bladder complications following his cases of typhoid fever, then by these combined means gall-bladder surgery will be materially lessened.

Tyson[1] states, "perhaps the most common cause of gall-bladder inflammation is the typhoid bacillus, the next most common cause is the colon bacillus."

Murphy[2] says, "The most common etiologic factor of gall-stones is the typhoid bacillus."

Deaver[3], "The infections of the biliary tract by the typhoid and para-typhoid bacilli are daily becoming of greater importance."

In the future neglect along these lines will render the physician culpable.

At present we have to deal with many cases of neglect, and gall-bladder surgery is in demand.

Cholecystostomy is now the operation of choice in majority of cases with most surgeons, the rubber drainage tube being properly dressed and invaginated, and gall-bladder dropped back into the abdominal cavity. The latter procedure in times past was considered dangerous but now practiced by the best surgeons except occasionally in cases of severe infection.

Immediate closure of gall-bladder without drainage and dropping back into abdomen formerly practiced now has but few indications.

In doing other operations in the abdomen, if stones are found in the gall-bladder, giving no symptoms and no special disease of gall-bladder, then cholecystendysis may be done but cholecystostomy is safer.

Kocher[4] favors this operation more than most authors.

Indications for cholcystostomy are well expressed by the Mayos[5], "It is indicated in all cases in which the gall-bladder is not seriously damaged and in which the removal of the calculi will leave the cystic duct free so there will be no further trouble from inefficient drainage, also in connection with disease of the common duct, and in all cases where the ducts of the liver are infected."

Operations during active severe inflammation of the common duct should be avoided, if possible, especially cutting into common duct, as death rate is very high, due to absorption of infection by the lymphatics.

Infection with impaction of stone in common duct, Kehr[6] lost 90 per cent. of cases operated on during the acute attack.

If operation is done it should be cholecystostomy and wait later for removal of stone, this is safer for most surgeons, but Deaver[7], advises immediate operation.

Of late considerable discussion has occurred as to indications for cholecystectomy. Much depends on the function of the gall-bladder and its ability to perform that function.

Deaver[8] "firmly believes that the gall-bladder has important functions to perform, of which the chief is to equalize the pressure of bile in the ducts and to secrete a mucous which dilutes the bile, and it acts to some extent as a storage for bile during long intervals between meals."

Mayo[9] "claims the function of the gall-bladder is probably to take the tension from the common and hepatic ducts and the ducts of the pancreas. It also secretes mucous, which mixed with the bile tends to reduce pancreatic and other complications, that it acts as a storage for bile is erroneous."

It is obvious that if the gall-bladder is so diseased or structure is so changed as to destroy its function, then cholecystectomy is indicated.

"[10]In some instances, a partial removal will leave a healthy portion that will functionate, then partial cholecystectomy is indicated."

Indiscriminate removal of the gall-bladder is not justifiable.

The borderline between cholecystectomy and -tostomy in some cases is difficult to decide. Such cases require discrimination and judgment.

Mayo[11] claims, "Cholecystectomy has a slightly higher death rate than cholecystostomy and ultimate results no better under similar conditions."

Deaver[12] is more radical and advises cholecystectomy in a wider range of cases.

Crile [13] describes what he calls cholecystitis obstruction cycle, as follows: "If the mucous membrane of the gall-bladder be gangrenous, if there be a stone imbedded by ulceration in the cystic duct; if the wall of the gall-bladder be thickened by scar tissue as a reaction to infection, and if there be no bile in the gall-bladder, these conditions will usually be followed by recurring obstruction and infection."

He admits that operation during acute infection with obstruction in common duct, death rate is higher.

Crile[14] advises against drainage of common duct, as it causes traumatism and stricture, urges "sharp dissection in bloodless white hair line." "Clean incision and immediate suture of common duct and, if drainage is needed, utilize gall-bladder, the operation being carried out under anoci-tech-technique.[15]"

He claims much of the bad results in common duct surgery is due to traumatism of the nerves affecting function of the liver, leakage of drainage, and infection, which are obviated by his method and technique.

Mumford[16] presents a condensed summary of surgery of the bile passages, as follows:

1. Remove stones.
2. Remove, so far as possible, all disorganized, degenerated, and permanently crippled tissue.
3. Drain.

As between cholecystostomy and cholecystectomy, he gives three cardinal rules:

Perform cholecystostomy:

(a) When gall bladder and ducts, though containing stones, are not crippled by inflammation, that is, when they are not markedly stenosed, thickened, twisted, or contracted.

(b) When acute inflammation exists, with or without the presence of stones. Acute inflammation demands thorough drainage.

(c) When the common duct is obliterated by unremovable malignant disease.

For cholecystectomy he gives two important indications:

(d) Disease crippling the gall-bladder.

(e) Disease crippling the cystic duct."

It is evident from the above opinions that there is no established rule for deciding between cholecystostomy and cholecystectomy. The guiding principle should be to preserve the gall-bladder and cystic duct so long as they can perform their functions without serious risk to the patient or be used to relieve other more serious conditions.

We must not lose sight of the circumstances in which the gall-bladder may be used in cholecystenterostomy, for drainage in cases of emergency in acute infections with obstruction of common duct, and also in the prevention and relief of hepatic and pancreatic complications.

Deaver[17] now advises cholecystectomy in diseases of the pancreas where he formerly advocated cholecystostomy, believing the chronic infection of the gall-bladder keeps up the foci of infection and that the drainage of the gall-bladder is not permanent.

When the gall-bladder has been destroyed or removed and a part of the common duct also destroyed an ingenuous operation has been done by Mann[18] of Minneapolis, in which he used a rubber tube in place of common duct in doing an hepatico duodenostomy, forming a channel from the junction of the hepatic ducts into the duodenum. For details of operation see reference eighteen.

Most authorities agree that the irritation from gall-stones form the primary basis for most cases of malignant disease in this region and that their removal is a preventive measure.

Moynihan[19] places gall-stones as the cause of 95 per cent.

Kocher[20] states, "We do not know of any case of malignant disease developing in a more or less healthy gall-bladder after removal of stones."

Deaver[21] "claims primary carcinoma of the gall-bladder and bile ducts is much more common than formerly suspected. It is found in about 5 per cent. of all cases of carcinoma. He quotes Shroder as stating that 14 per cent. of all cases of gall-stones eventually suffer from carcinoma of the biliary apparatus."

Mayo[22] finds 4 per cent. of cancer in 1800 operations upon gall-bladder and bile passages, 75 per cent. in gall-bladder and 25 per cent. in bile ducts.

As to indications and contraindications for gall-bladder surgery, the radical surgeon may find food for thought by reading the article on Disease of the Liver Bile Passages and Gall-bladder in Fochheimer-Therapeutics of Internal Disease, Vol. 3.

A point of practical importance is the Typhoid Carrier from the relation they bear to the prevention of typhoid and the treatment of the individual carrier.

Deaver[23] gives the estimate of 1.7 to as high as 5 per cent. of the total number of typhoid cases, claiming there are carriers that have not had typhoid fever and reports (7) cases demonstrated by operation, finding the typhoid bacillus in gall-bladder. He recommends cholecystectomy in these cases if sero-therapy and other means of treatment fail.

Here it is the responsibility of the internist again comes in emphasizing his duty, to prevent gall-bladder, renal and other complications, also, if possible, the typhoid carrier.

This opens up a field beyond the scope of this paper, however, I wish to give a few general suggestions.

The serums, and more especially the sensitized bacterins, give much promise of usefulness.

Shattuck[24] reports thatt Moffit has collected 8 cases of typhoid carriers cured by antityphoid vaccination."

We should not lose sight of the work of Crowe in demonstarting the effect of hexamethylenamine in disinfecting the gall-bladder and its known effect on the urine.

Septic conditions are factors in the production of some cases of gall-bladder disease as well as other destructive changes requiring surgical work.

The future of the internist along the line of prevention and treatment of infections is bright and promising. The proper use of bacterins, internal medication, hygienic measures and diet will accomplish marked results.

Along the line of therapeutics I predict a wide range of usefulness for iodine, internal as well as external; it will, if properly used, be recognized as an antitoxin and stimulator of the ductless glands of the body; quinine, in sufficient doses, short of its hemolytic action, will be recognized as an inhibitor of germ development, while hexamethylenamine will, in proper doses associated with necessary acid condition, be recognized as a germ destroyer of considerable value.

Since writing this paper I see Vaughn[25] in his address on Parenteral Protein Digestion, states, "Quinine delays the action of bacterial proteases; this, in part at least, may explain its action."

Lewis and Krauss[26] find, "In examining the relative distribution of iodin-yielding drugs in healthly and diseased tissues, respectively, they ascertained that tuberculous tissue derived from animals to which no iodin preparation had been knowingly administered may contain amounts of iodin very appreciably higher than the normal content of the same animal. For example, the tuberculous cornea of rabbits inoculated in one eye with tubercle bacilli was found to have as much as 2.67 mg. of iodin per gram of dry tissue, whereas the healthy cornea was iodin-free."

I am not a therapeutic nihilist and have but little patience with the scientific internist that would rather let his patient die scientifically than cure him empyrically with remedies often proven useful by clinical experience.

1. Journal A. M. A., April 24, 1914.
2. Murphy: Surg. Clinics, Vol. 2, No. 6, page 1049.
3. Deaver: Surg. Upper Abd., Vol. 2, page 17.
4. Kocher: Operative Surg., Vol. 2, page 539.
5. Keen: Surg., Vol. 3, page 1016.
6. Murphy: Clinics, Vol. 1, No. 3, page 418.
7. Deaver: Surg. Upper Abd., Vol. 2, page 119.
8. Deaver: Surg. Upper Abd., Vol. 2, page 115.
9. Keen: Surg., Vol. 6, page 601.
10. Keen: Surg., Vol. 6, page 603.
11. Keen: Surg., Vol. 6, page 602.
12. Deaver: Annals Surg., June, 1914; page 846.
13. Crile: Surg., Gynec. and Obs., Apr., 1914; page 429.
14. Crile: Jour. A. M. A., May, 1914; page 1373.
15. Crile & Clower Anoci-Association, page 136.
16. Mumford: Practice of Surg., page 174.
17. Deaver: Annals Surg., June, 1914; page 846.
18. Mann, Arthur T.: Surg., Gynec. & Obs., Mar., 1914; page 327.
19. Moynihan: Abdominal Operations, page 537.
20. Kocher: Operative Surg., Vol. 2, page 533.
21. Deaver: Surg. Upper Abd., Vol. 2, page 217.
22. Keen: Surg., Vol. 3, page 1005.
23. Deaver: Surg. Upper Abd., page 24.
24. Forchheimer: Therapeutics Inter. Med., Vol. 2, page 6.
25. Vaughan: A. M. A. Jour., Aug. 1, 1914; page 366.
26. Editorial: A. M. A. Jour., Aug. 1, 1914; page 409.

Empire Life Bldg., Atlanta, Ga.

